# A Qualitative Study Exploring Barriers and Facilitators of Enrolling Underrepresented Populations in Clinical Trials and Biobanking

**DOI:** 10.3389/fcell.2019.00074

**Published:** 2019-04-30

**Authors:** Terry C. Davis, Connie L. Arnold, Glenn Mills, Lucio Miele

**Affiliations:** ^1^Department of Medicine, Feist-Weiller Cancer Center, LSU Health Sciences Center Shreveport, Shreveport, LA, United States; ^2^Stanley S. Scott Cancer Center, LSU Health Sciences Center New Orleans, New Orleans, LA, United States

**Keywords:** biobanking, genomics, clinical trials, underrepresented populations, health literacy

## Abstract

Disparities exist in enrollment in clinical trials and biorepositories among adults with low socioeconomic status, racial and ethnic minority groups and individuals who live in rural areas. Diverse participation is necessary to identify the most effective treatments in different groups. The purpose of this study was to use qualitative methods to identify factors that may affect the likelihood that members of underrepresented groups choose to participate in clinical trials and/or biobanking. We conducted 14 focus groups and seven telephone interviews in urban and rural areas of Louisiana to: (1) identify barriers and facilitators to participation; and (2) elicit input in crafting clear, culturally appropriate language and recruitment strategies. Of 103 participants, 25 were safety-net healthcare providers, 18 were primary care or oncology clinic patients, and 60 were members of social and faith-based groups. Patients and community participants were English-speaking, 79% were African American, 81% were female and 24% lived in rural areas. Barriers to participation identified were lack of knowledge about clinical trials and biobanks; limited specific information and access to participation, trust and privacy concerns about clinical trials and biobanking Facilitators included: altruism, high interest in medical research particularly studies that might benefit them or their families; plain language, culturally appropriate information; convenient access to studies; and input of a trusted provider. In addition, all primary care providers were interested in having clinical trial options available for their patients but did not have time to search for available trials. Results of this study can inform the development of education materials and strategies to increase participation of underrepresented groups in clinical trial and biobanking.

## Introduction

The National Institutes of Healthdefines precision medicine as “an emerging approach for disease treatment and prevention that takes into account individual variability in genes, environment, and lifestyle for each person” (Genetics Home Reference at National Institutes of Health, 2018). Precision medicine offers the potential for improved prevention and treatment, avoidance of adverse events, and rational selection of medications (National Cancer Institute at National Institutes of Health, 2017). However, the implementation of precision medicine is critically dependent upon access to adequate data from study populations that reflect the diversity of society in terms of genetics, socioeconomic status, and lifestyles (The Precision Medicine Initiative at Obama White House Archives, 2016). Disparities exist in recruitment, retention, and trust in clinical trials and biorepositories among individuals who have low socioeconomic status, limited literacy, those who belong to racial, ethnic minority groups, and people who live in rural areas (George et al., 2014; Wolf et al., 2016). Participation of racial and ethnic minorities and underserved populations in clinical trials is a critical link between scientific innovation and improvements in healthcare delivery and health outcomes (Simon et al., 2014).

Despite recent emphasis of improving diversity, the National Cancer Institute Center to Reduce Cancer Health Disparities’ recent survey of their Research Network’s biorepositories found only one-tenth of their specimens are from non-white patients (Friedman et al., 2013). Moreover, less than 5% of cancer patients are enrolled in a clinical trial and less than 10% of these are minorities (Chen et al., 2014). Within all NIH clinical trials at United States sites, African American enrollment is only 10% (U.S. Food and Drug Administration, 2017). Additionally, despite well-documented health disparities in rural areas, few studies are conducted among rural populations (Baquet et al., 2006; Friedman et al., 2015). In the last 5 years, only 3% of NCI’s Cancer Control and Population Sciences clinical trials have focused on rural populations (Kennedy et al., 2018). The Cancer Moonshot Task Force has called for steps to ensure that all Americans, including those who have limited resources and those that live far distances from major cancer centers have access to leading edge cancer treatment, prevention, screening approaches, and research (The Report of the Cancer Moonshot Task Force at Obama White House Archives, 2016). Greater understanding of barriers and facilitators to underrepresented populations participation in clinical trials and biobanking can improve recruitment and retention strategies and better inform future studies (Jones et al., 2009; George et al., 2014).

The Precision Medicine Initiative, through the “All of Us” Research Program, plans to enroll approximately one million participants who reflect the diversity of America (All of Us Research Program Initial Protocol, 2018). These individuals are asked to give consent for the banking of their biological specimens (blood cell populations, proteins, metabolites, RNA, DNA for genotyping and whole genomic sequencing when cost permits) all linked to their electronic health record (EHR). If properly implemented, this research program has the potential to greatly increase the tools available to the clinical research community to identify informative data patterns, new markers of genetic risk of disease or adverse events and new diagnostic and/or predictive biomarkers. Diverse enrollment will be vital to the success of the program. The Beta phase launched in 2017 exposed the need for messages to be simpler. Privacy, trust, transparency, data quality and integrity and responsible return of genomic data to participants are actively being discussed (All of Us Research Program Initial Protocol, 2018).

The rapidly developing field of precision medicine and the growing number of diagnostic genomic tests are resulting in a widening gap between the knowledge base available to researchers and the understanding of genomics by patients’ primary care providers and the public. The magnitude of this divide may be more significant among providers in safety-net clinics and among low income, rural and minority populations, who are currently underrepresented in clinical trials. This may limit the ability of these groups to understand, access and participate in state-of-the-art research and treatment.

We conducted a pilot study to: (1) to identify factors that may influence the decision of members of underrepresented groups to participate in clinical trials and/or biobanking and (2) to elicit their input in crafting clear, culturally appropriate language and recruitment strategies.

## Methods

We conducted a small qualitative study in January – July 2017 in four towns in Louisiana to assess barriers and facilitators influencing understanding and participation to clinical trials and biobanking. We conducted 14 focus groups with English-speaking patients and providers in safety-net primary care and oncology settings and in social, faith-based as well as Parkinson’s and Alzheimer’s caregiver support groups. Additionally, we conducted seven structured individual telephone interviews with caregivers unable to meet at time of focus groups. IRB approval was obtained from the LSUHSC Institutional Review Board. Two members of the research team, who are trained focus group facilitators, explained the study and consented all participants. Patients and community group participants were paid $35 for their time and providers were paid $100.

## Participant Recruitment

A director or leader at each clinical and community site assisted with recruitment of a convenient sample of patients and providers by distributing flyers and announcing the focus groups at group meetings. They also scheduled the focus groups on-site at a convenient time for participants. Demographics are listed in Table 1. Patients and community participants were recruited from a university cancer center, an academic safety net medicine clinic, and an urban and rural Federally Qualified Health Center (FQHC), two African American sororities, an African American church, a rural and two urban Council on Aging sites, a Volunteers of America Alzheimer’s Family Caregiver support group and a Parkinson’s Caregiver support group. Individual phone interviews were conducted with caregivers who could not meet the day scheduled for the group. The same moderators guide was used for the focus groups and individual interviews. All participants were representatives of low income, minority or rural groups that are underrepresented in clinical trials. Providers were recruited by the medical director at each clinic.

**TABLE 1:** Participant demographics.

	(*n* = 103)		
	
	Clinic patients	Community group	Clinic providers
	*n* = 18	*n* = 60	*n* = 25
**Race**			
African American	12 (67%)	50 (83%)	8 (32%)
Hispanic	1 (1%)	0	0
White	5 (28%)	10 (17%)	17 (68%)
**Gender**			
Female	13 (72%)	53 (88%)	15 (60%)
Male	5 (28%)	7 (12%)	10 (40%)

*The 7 individual interviews were all with family caregiver support group members.*

## Development of Moderators Guide for Focus Groups and Individual Telephone Interviews

The research team developed two semi-structured, open-ended focus group guides; one for providers and one for patients and community participants. Both guides were based on a review of the literature and discussions with biobanking and genomics experts and providers conducting clinical trials. Both guides included open-ended questions about the extent of awareness, knowledge, experience trust and acceptance of biobanking, genomics and clinical trials. The guide also included questions on barriers and facilitators to communication and access to clinical trials and biobanking (Table 2).

**TABLE 2:** Moderators guide for patient genomic focus groups.

We want to learn what people think about participating in medical research studies and allow their tissue to be sent to biobanks.
Today we want to learn what you think about these new possibilities for more personalized health care treatments that can be learned from research involving use of a wide variety of people’s blood or body tissue.
• We are not trying to enroll you in a genomics research study – we just want your thoughts about these new kinds of studies.
• We are interested in your thoughts and experiences about participating in medical research studies
• We want to hear your thinking, questions, concerns, beliefs and experiences.
** (1) Have you ever been in a research study?**
a. Probe: How did you find out about it? Did a doctor or nurse ask you if you wanted to participate?
b. Have you ever been asked to be in one?
** (2) Have you ever heard of genomics research**? If yes:
a. What have you heard?
b. Would you like to know more about what genomics means and what it could mean for you?
**(3) Have you ever heard of biobanking?**
**a.** What does this mean to you?
**b.** What does taking a sample of your tissue or blood mean to you?
**c.** What questions do you have about researchers using your tissue or blood and putting them in a biobank?
**(4) What questions/concerns do you have participating about this kind of research?**
**a.** What would you want to know before you agreed to participate in a study that used your cells / tissue?
**(5) What information would you like about studies that use people’s genes or tissue samples?**
**(6) How do you think people need to get this information?**
**a.** Probe: doctor/nurse pamphlet, internet
**(7) What does written information need to say?**
a. Probe: What terms are most useful or helpful or confusing?
**(8) Where do you get health information?**
a. Probe: Doctor’s office, internet, health magazines, television
**(9) Would you be interested in participating in biobanking?**
**a.** Probe: What would be barriers to participating? What would your concerns be?
**(10) Would you be interested in participating in a clinical trial?**
**a.** Probe: What studies would you be most interested in – diseases that affect your family or yourself?

## Procedure

Two of the authors, trained qualitative researchers, conducted focus groups and individual telephone interviews. One author and one research assistant took notes. Groups averaged 75–90 min in length, and telephone calls lasted 60 min; all were audio recorded and followed the moderator’s guide and semi-structured interview format focus group. All sessions were recorded, and verbatim notes were taken by a member of the research team. The notes were organized according to the semi-structured interview format.

## Data Management and Analysis

Interviews and focus groups were transcribed verbatim and verified for accuracy. A research assistant coded transcript using NVivo software (QSR International Pty Ltd., 2012). The constant comparative method of grounded theory was used to identify developing themes. With regard to barriers and facilitators, six main themes were identified: (1) knowledge and awareness; (2) understanding of clinical trial and biobanking information and terminology; (3) trust and privacy; (4) interest and attitudes, (5) strategies to increase enrollment of underrepresented populations; (6) and recruitment suggestions.

## Results

A total of 103 participants were enrolled in the study. Twenty-five were health care providers, 18 were clinic patients, and 60 were community participants; 24% lived in rural communities with populations less than 10,000. Patients and community participants ranged in age from 50 to 80 and were mostly female (81%), and African American (79%). Of the 25 providers, ten were physicians, five nurse practitioners, seven cancer research associates and three were behavioral health professionals. Providers were 32% African American and 60% female.

Participants noted several barriers and facilitators to participating in clinical trials and biobanking. Barriers included lack of knowledge, limited understanding of specific information, mistrust and access problems (transportation). Facilitators include altruism, desire to find cure for themselves or their families, to help improve treatments for future generations, particularly their grandchildren or to be a part of something big and positive. These are reported more specifically in the focus group themes below.

## Barriers

### Knowledge and Awareness

Both patient and community group participants were aware of clinical trials and genomic studies from watching TV. Most had seen ads for cancer treatments centers and for (23andMe, 2018) (^©^2019 23andMe, Inc.). In general, there was limited knowledge about medical research or genomic studies. Although two primary care patients and two oncology patients had been enrolled in a clinical trial, there was in general a lack of knowledge about specific trials or where to find out about them. No participant had heard of biobanks. After the term was explained rural participants wanted to know: “*Where is the bank located?”*

Very few patients, family caregivers or providers said they looked for clinical trials on the internet or social media. When probed about looking for appropriate clinical trials, only one participant, a member of the Parkinson’s caregiver group had looked for studies on the internet. Most had not thought about looking for trials. Participants preferred to learn about studies from a trusted source-most commonly their physician.

### Understanding of Clinical Trial and Biobanking Terminology

Participants were not familiar with terms commonly used in clinical trials and biobanking. The investigators asked participants to suggest plain language terms to improve understanding and acceptance. Suggestions included: rather than use the term clinical trial they suggested *study or medical research*. *“Everyone understands what a study is.”* Genomics – sounded intimidating, alarming “*it sounds scary.”* Even though most participants had heard the terms DNA and genetics the term *genomics* did not mean anything to most. After the term was explained, several participants were still uncertain what it was, or did not seem very interested. For biobanking, participants suggested a more concrete explicit explanation*, “your blood or tissue will be stored in a bank*.”

### Trust and Privacy

All safety net primary care and cancer patients reported they trusted their physician. This was particularly true in rural community clinics, “*I always want to know what doc thinks, whatever he says I will do.”* The cancer Clinical Research Associates (CRAs) reported that people who have cancer are usually receptive to clinical trials, “*patients do whatever their doctor wants them to do, but it is also dependent on the relationship the patient has with the research coordinator, and on what is happening with the patient that day and what most important to them*.” CRAs said sometimes after enrolling a patient an adult child will call and say, *“I don’t want my mother put on a study*.” Concerning biobanking, CRAs reported that cancer patients don’t like to be stuck an extra time*, “it’s usually fine if they are already having blood drawn or if they a have a port.”* They said most of their patients are receptive to biobanking but “*in the last few years highly educated people have become more resistant to the idea of biobanking*. *They don’t want tests done on their tissue or blood that they don’t know about.”* In general people wanted to know*: “What are you going to do with my blood sample? How much information about “me” are you going to keep? What is it going to be used for? Who is going to see my information? Will it hurt me getting a job or insurance?”* Despite being told of protections; some participants were still concerned about privacy and if the information would be used as a barrier to jobs or insurance.

African American participants strongly suggested messages regarding clinical trials and biobanking needs to incorporate all races and ethnicities. They suggested saying “*All people are needed for studies to improve treatments and find cures*. *Targeting African American recruitment makes it appear suspicious*.” Some African American patients and providers in rural clinics and participants in African American social and faith-based groups mentioned the Tuskegee study might be a barrier to African Americans enrolling in medical research studies or biobanking. African Americans in community groups were less trusting of clinical trials than African American patients. African American patients in rural and urban clinic groups reported more trust in their long-time health care providers.

Some African American community participants reported they did not feel close to their or their family’s physicians and they did not feel doctors had been helpful, “*he never told us about any studies or gave us much helpful information about my mother’s Alzheimer’s.”* One African American church group participant said she thought doctors had a mindset that “*once you are 80 you lived long enough.”* However, almost all participants trusted the accuracy of health information from their physician. African American women in rural and urban community groups differed in their trust of health information and recommendations from their pastor, rural participants said they didn’t want to get health information from their pastor, “*you never know how much education some of these preachers have.”*

### Transportation

Both providers and patients said transportation was a barrier to participation. This was particularly true for low-income urban patients and for those living in rural areas. Rural providers said their patients had to go to an academic health center if they wanted to participate in a clinical trial. Providers and patients liked the idea of a mobile health van that could come to their clinic for study visits. They felt this would improve access to trials and biobanking. Several medical residents in the safety-net hospital medicine clinic mentioned convenience, “*It’s hard enough for our patients to keep medical appointments without having to come back to participate in a clinical trial.”*

## Facilitators

### Interest and Attitudes

Almost all participants were interested in clinical trials but not aware of studies or how to find out about them or enroll. Most FQHC patients were willing to participate but only two had been asked and both of these participated one in a study on disparities and the other in a diabetes study recommended by a specialist at an academic health center. Community participants in caregiver groups were particularly interested in learning about studies focused on diseases their family had. Patients in low income clinics and community participants were also interested in studies of diseases they or their families had, *“everybody knows someone who has had cancer treatment.”* Community clinic providers and medicine residents were highly interested in clinical trials that would be appropriate for their patients. Primary care patients said they would enroll in genomic studies and biobanking to benefit others, particularly their grandchildren, even if there was no benefit to them. These sentiments were particularly strong among rural patients.

All urban and rural safety net providers who participated were interested in being involved in clinical trials. However, they lacked the time to identify studies and explain them to patients. No primary care provider had looked for clinical trials appropriate for his or her patients on the internet. To increase participation of rural and minority patients, they suggested on site in-services or webinars to give them information about clinical trials available for their patients and biobanking. For specific studies, they requested brief plain language information with talking points and a card they could give patients with a name and number to call for more information. *I need to know basic information like how the study would affect their patients, the time commitment, how long they would be in the study and where they would have to go to participate*. Providers suggested that researchers need to remind people: “*You are giving back. Even though this may not help you, it will help others in the future who have your disease or other diseases like diabetes, heart disease, cancer. This may help your children or your grandchildren. You can help save lives.”* CRAs often reminded patients *that nothing happens in cancer treatment that isn’t the result of prior research studies. Many people have participated in trials before them to get us where we are in cancer treatment currently.* Community physicians also suggested information about clinical trials and biobanking would be helpful to have for talks they are asked to give to groups in their community.

### Transportation

Providers and patients liked the idea of a mobile health van that could come to their clinic for study visits. They felt this would improve access to trials and biobanking.

### Strategies to Increase Enrollment of Underrepresented Populations

Safety-net patient providers, patients and minority groups said information on clinical trials and biobanking would be most effective if it came from a trusted provider, *“If my doctor recommended it, I would participate*.” Some patients suggested a poster in clinic waiting room to prompt them to ask their physician about the study. Billboards, posters in the grocery store or newspaper or TV ads seemed somewhat suspicious, “*If there was a flyer in a grocery store or post office, I wouldn’t trust it.”* Participants were more trusting of articles in health system publications or trusted agencies such as the Council on Aging or AARP.

## Discussion

Our ability to analyze human biospecimens through genomics and other technologies has grown exponentially over the last two decades. At the same time, advances in electronic health records and computational “big data” technology have enabled the construction and analysis of vast clinical datasets to seek patterns predictive of clinical outcomes. However, without a diverse representation that reflects the population at large, precision medicine research and implementation will exacerbate health disparities rather than diminishing them.

The barriers and facilitators to enrollment in clinical trials and biobanking among low income, African American and rural groups we identified were similar to those reported in other focus groups and individual interviews with minority, rural and urban participants across the United States (Ford et al., 2005; Baquet et al., 2006; Howerton et al., 2007; Jones et al., 2009; Streicher et al., 2011; Halverson and Ross, 2012; Sanderson et al., 2013; Friedman et al., 2015; Heredia et al., 2016). One of the main barriers was lack of knowledge and minimal understanding about medical research as well as lack of awareness of biobanking. Few participants had ever enrolled in a clinical trial or had of a family member participate in one. As in previous studies, we believe this may stem from a general lack of education about biobanks, genomic research and clinic trials among underrepresented groups and minimal outreach by researchers (Ford et al., 2005).

As in other studies of minority participants, we found trust of medical research was a barrier (Ford et al., 2005; Streicher et al., 2011; Friedman et al., 2015). However, in our study as in others, patients in safety-net community clinics reported a great deal of trust in their primary care provider and said they would likely participate in a study or biobanking if their nurse practitioner or physician recommended it. Freidman also found focus group participants said they were more likely to participate in a clinical trial if their physician recommended it (Friedman et al., 2013), it seems trust in one’s own provider may be a potential mediating factor in alleviating mistrust of medical research among some underrepresented groups. As with our rural patients, Friedman et al. (2015) found transportation was also a barrier to participation. Our rural providers and patients also suggested a health van from the academic cancer center might facilitate participation.

As in other studies (Dang et al., 2014; Heredia et al., 2016), we found facilitators were altruism and a desire to advance medicine. Our participants spoke of helping future generations, particularly grandchildren, even if it would not benefit them. Our participants wanted health information to come from their health providers, however, only one had a primary care doctor who recommend a clinical trial.

A review of 18 studies examining provider-related factors influencing recruitment of underrepresented populations to clinical cancer trials (Dang et al., 2014) found some similarity and some differences with the findings of our providers. As in our study, lack of provider awareness of clinical trials, available protocols and concern about patient cost were barriers. Unlike other studies, our providers did not express attitudinal barriers relating to patient adherence to study protocol, misunderstanding of research and data collection cost. In our study, community providers expressed interest in enrolling patients and the only barriers mentioned were time and staff skill and capacity in consenting and enrolling patients. They also mentioned patient participation barriers such as transportation and suggested mobile health vans could facilitate access and participation.

Understandable and accessible communication was part of our focus. Other studies mentioned lack of education and outreach as barriers to engaging underserved populations. In focus groups across the country, (Streicher et al., 2011; Dang et al., 2014) found Hispanic participants suggested terms like *library* and *warehouse or storage* rather than bank. Our ability to communicate and describe these complex clinical research projects to the prospective participants who would most benefit from, and to their primary care providers, has yet to catch up to the technological progress. Our results indicate a wide communication gap between those who design and execute precision medicine research and prospective participants, especially those who live under disadvantaged socioeconomic conditions and/or in areas distant from academic health centers.

Researchers often assume patients and community providers know more than they do about clinical trials, understand the jargon and know how to access information. Plain language, culturally appropriate, accessible information on clinical trials, precision medicine, genomics and biobanking for community clinic providers, their patients and the public is needed. Information on precision medicine, clinical trials and biobanking needs to be easy to find, honest, transparent, culturally appropriate, understandable and actionable. To recruit more people, particularly those underrepresented in biomedical research, personal “high-touch” outreach by researchers and coordinators is needed to community providers and trusted agencies. A focus needs to be on providers in Federally Qualified Health Centers that are located in underserved areas nationally and serve over 27 million patients (HRSA Health Center Program, 2018). These community physicians want basic information about clinical trials to inform their patients. Like Jones et al. (2009), our experience conducting the focus groups in rural and urban clinics and agencies that serve underserved populations point to the importance of researchers visiting such clinics and agencies and creating trusting relationships with leaders. This then leads to the opportunity to help bridge a trusting relationship with potential participants who are served by these agencies and have trust in the clinic providers and/or agency staff.

### Limitations

The study limitation include that the study was qualitative and focused on a limited number of people underrepresented in clinical trials and genomic studies. The study was limited to English- speaking patients, providers in one state. However, one fourth of the participants lived in rural communities, and the majority were African American. It is noteworthy that a vast majority of our participants were female. Gender differences in health care needs and utilization are well-characterized in the United States (Cameron et al., 2010). It is possible that men from under-represented minorities may require differently tailored messages to enhance interest in clinical trial and/or biobanking participation.

## Conclusion and Recommendations

Our study suggests that the content of plain language biobanking information and plan to disseminate the information needs the input of the public, minority groups and low-income community patients and providers. Investigators do not and, in many instances, cannot know how bio specimens will be used in the future, but investigators need to work with the public to craft honest, transparent messages about how the biospecimens and the clinical data associated with them can and cannot be used. Future qualitative research also needs to be done, with a wider range of participants from ethnic and minority populations, those with ESL and from a wider range of rural areas nationally. Finally, the notion of “return of value,” i.e., reporting individual results from precision medicine studies to the participants themselves and aggregate results to the public at large, is gaining favor as a way to democratize the results of precision medicine research and provide individual participants information they could use to manage their health. The “All of Us” Precision Medicine Initiative, for example, has proposed returning value to participants by reporting to them actionable results from genomic testing and other measurements (All of Us Research Program Initial Protocol, 2018). In order to maximize the value of this information to individuals and the public, researchers need input on how people would want to receive this information in an acceptable, understandable and useful form. Within its limitations, our study supports the following recommendations:

(1) Messages about clinical trials and biobanks need to be crafted with input from prospective participants.
(2) Primary care providers may have a key role in improving clinical trial participation as trusted intermediaries between the research community and potential research participants. However, they must be equipped with culturally sensitive, appropriate material that is as self-explanatory as possible to inform potential participants. Given their limited time availability, they cannot be expected to search for clinical trials and explain them to their patients without assistance.
(3) Visiting clinics and agencies serving low income rural and minority populations helps researchers create trusting relationships with providers and staff and can help bridge a trusting relationship with potential participants.
(4) Transportation is a barrier to participation, and innovative recruitment strategies and study designs that make studies more accessible to participants are needed.
(5) Interpersonal interactions remain critical in establishing trust in medical research, and technology-driven substitutes are unlikely to perform well among under-represented groups.

## Ethics Statement

This study was carried out in accordancewith the recommendations of “LSU Health Sciences Center Institutional Review Board for the Protection of Human Research Subjects.” This was a minimal risk study using focus groups and a waiver of written consent was requested and granted. All participants were given a consent letter. All subjects gave oral informed consent in accordance with the Declaration of Helsinki. The protocol was approved by the “LSU Health Sciences Center Institutional Review Board for the Protection of Human Research Subjects.”

## Author Contributions

CA, TD, LM, and GM contributed to the conception and design of the study and performed the qualitative data analysis. CA organized the focus group notes. TD wrote the first draft of the manuscript. CA and LM wrote sections of the manuscript. All authors contributed to the manuscript revision, read, and approved the submitted version.

## Conflict of Interest Statement

The authors declare that the research was conducted in the absence of any commercial or financial relationships that could be construed as a potential conflict of interest.

## References

[B1] 23andMe (2018). *DNA Genetic Testing & Analysis - 23andMe. How it Works – 23andMe.* Available at: https://www.23andme.com/ (accessed February 25, 2019).

[B2] All of Us Research Program Initial Protocol (2018). *All of Us Research Program Initial Protocol.* Available at: https://allofus.nih.gov/about/all-us-research-program-protocol (accessed November 15, 2018).

[B3] Baquet C. R. Commisky P. Daniel Mullins C. Mishra S. I. (2006). Recruitment and participation in clinical trials: socio-demographic, rural/urban, and health care access predictors. *Cancer Detect. Prev.* 30 24–33. 10.1016/j.cdp.2005.12.001 16495020 PMC3276312

[B4] Cameron K. A. Song J. Manheim L. M. Dunlop D. D. (2010). Gender disparities in health and healthcare use among older adults. *J. Womens Health* 19 1643–1650. 10.1089/jwh.2009.1701 20695815 PMC2965695

[B5] Chen M. S. Lara P. N. Dang J. H. T. Paterniti D. A. Kelly K. (2014). Twenty years post-nih revitalization act: renewing the case for enhancing minority participation in cancer clinical trials. *Cancer* 120 1091–1096. 10.1002/cncr.28575 24643646 PMC3980490

[B6] Dang J. H. T. Rodriguez E. M. Luque J. S. Erwin D. O. Meade C. D. Chen M. S. Jr. (2014). Engaging diverse populations about biospecimen donation for cancer research. *J. Commun. Genet.* 5 313–327. 10.1007/s12687-014-0186-0 24664489 PMC4159470

[B7] Ford J. G. Howerton M. W. Bolen S. Gary T. L. Lai G. Y. Tilburt J. (2005). *Knowledge and Access to Information on Recruitment of Underrepresented Populations to Cancer Clinical Trials: Summary.* Rockville, MD: Agency for Healthcare Research and Quality. 10.1037/e439572005-001 PMC4781635 15989377

[B8] Friedman D. B. Bergeron C. D. Foster C. Tanner A. Kim S.-H. (2013). What do people really know and think about clinical trials? A comparison of rural and urban communities in the south. *J. Community Health* 38 642–651. 10.1007/s10900-013-9659-z 23468319

[B9] Friedman D. B. Foster C. Bergeron C. D. Tanner A. Kim S.-H. (2015). A qualitative study of recruitment barriers, motivators, and community-based strategies for increasing clinical trials participation among rural and urban populations. *Am. J. Health Promot.* 29 332–338. 10.4278/ajhp.130514-qual-247 24670073

[B10] Genetics Home Reference at National Institutes of Health (2018). *Genetics Home Reference at National Institutes of Health.* Available at: https://ghr.nlm.nih.gov/primer/precisionmedicine/definition (accessed October 24, 2018).

[B11] George S. Duran N. Norris K. A. (2014). Systematic review of barriers and facilitators to minority research participation among african americans, Latinos, Asian Americans, and Pacific Islanders. *Am. J. Public Health* 104 e16–e29. 10.2105/AJPH.2013.301706 24328648 PMC3935672

[B12] Halverson C. M. Ross L. F. (2012). Engaging african-americans about biobanks and the return of research results. *J. Community Genet.* 3 275–283. 10.1007/s12687-012-0091-3 22454259 PMC3461227

[B13] Heredia N. I. Krasny S. Strong L. L. Von Hatten L. Nguyen L. Reininger B. M. (2016). Community perceptions of biobanking participation: a qualitative study among Mexican-Americans in Three Texas Cities. *Public Health Genomics* 20 46–57. 10.1159/000452093 27926908 PMC5453816

[B14] Howerton M. W. Gibbons M. C. Baffi C. R. Gary T. L. Lai G. Y. Bolen S. (2007). Provider roles in the recruitment of underrepresented populations to cancer clinical trials. *Cancer* 109 465–476. 10.1002/cncr.22436 17200964

[B15] HRSA Health Center Program (2018). *Increasing Access to Care.* Available at: https://bphc.hrsa.gov/sites/default/files/bphc/about/healthcenterfactsheet.pdf (accessed October 5, 2018).

[B16] Jones R. A. Steeves R. Williams I. (2009). Strategies for recruiting african american men into prostate cancer screening studies. *Nurs. Res.* 58 452–456. 10.1097/nnr.0b013e3181b4bade 19918156 PMC2886148

[B17] Kennedy A. E. Vanderpool R. C. Croyle R. T. Srinivasan S. (2018). AN overview of the national cancer institute’s initiatives to accelerate rural cancer control research. *Cancer Epidemiol. Biomarkers Prev.* 27 1240–1244. 10.1158/1055-9965.epi-18-0934 30385495 PMC13577081

[B18] National Cancer Institute at National Institutes of Health (2017). *National Cancer Institute at National Institutes of Health.* Available at: https://www.cancer.gov/about-cancer/treatment/types/precision-medicine (accessed July 17, 2018).

[B19] QSR International Pty Ltd. (2012). *NVivo Qualitative Data Analysis Software; Version 10.*

[B20] Sanderson S. C. Diefenbach M. A. Zinberg R. Horowitz C. R. Smirnoff M. Zweig M. (2013). Willingness to participate in genomics research and desire for personal results among underrepresented minority patients: a structured interview study. *J. Community Genet.* 4 469–482. 10.1007/s12687-013-0154-0 23794263 PMC3773313

[B21] Simon M. A. de la Riva E. E. Bergan R. Norbecket C. McKoy J. M. Kulesza P. (2014). Improving diversity in cancer research trials: the story of the cancer disparities research network. *J. Cancer Educ.* 29 366–374. 10.1007/s13187-014-0617-y 24519744 PMC4029870

[B22] Streicher S. A. Sanderson S. C. Jabs E. W. Diefenbach M. Smirnoff M. Peter I. (2011). Reasons for participating and genetic information needs among racially and ethnically diverse biobank participants: a focus group study. *J. Community Genet.* 2 153–163. 10.1007/s12687-011-0052-2 22109822 PMC3186034

[B23] The Precision Medicine Initiative at Obama White House Archives (2016). *The Precision Medicine Initiative at Obama White House Archives.* Available at: https://obamawhitehouse.archives.gov/node/333101 (accessed July 16, 2018).

[B24] The Report of the Cancer Moonshot Task Force at Obama White House Archives (2016). Available at: https://obamawhitehouse.archives.gov/sites/default/files/docs/final_cancer_moonshot_task_force_report_1.pdf (accessed October 1, 2018).

[B25] U.S. Food and Drug Administration (2017). *Global Participation in Clinical Trials Report.* Available at: https://www.fda.gov/downloads/Drugs/InformationOnDrugs/UCM570195.pdf [accessed February 22, 2019].

[B26] Wolf R. L. Basch C. E. Zybert P. Basch C. H. Ullman R. Shmukler C. (2016). Patient test preference for colorectal cancer screening and screening uptake in an insured urban minority population. *J. Community Health* 41 502–508. 10.1007/s10900-015-0123-0 26585609

